# Sequence conservation analysis and *in silico* human leukocyte antigen-peptide binding predictions for the Mtb72F and M72 tuberculosis candidate vaccine antigens

**DOI:** 10.1186/s12865-015-0119-7

**Published:** 2015-10-22

**Authors:** Marie-Cécile Mortier, Erik Jongert, Pascal Mettens, Jean-Louis Ruelle

**Affiliations:** GSK Vaccines, Rue de l’Institut 89, 1330 Rixensart, Belgium

## Abstract

**Background:**

Requisites for an efficacious tuberculosis (TB) vaccine are a minimal genomic diversity among infectious *Mycobacterium tuberculosis* strains for the selected antigen, and the capability to induce robust T-cell responses in the majority of human populations. A tool in the identification of putative T-cell epitopes is *in silico* prediction of major histocompatibility complex (MHC)-peptide binding. Candidate TB vaccine antigen Mtb72F and its successor M72 are recombinant fusion proteins derived from Mtb32A and Mtb39A (encoded by *Rv0125* and *Rv1196*, respectively). Adjuvanted Mtb72F and M72 candidate vaccines were shown to induce CD4^+^ T-cell responses in European, US, African and Asian populations.

**Methods:**

Sequence conservation of Mtb32A, Mtb39A, Mtb72F and M72 among 46 strains (prevalent *Mycobacterium* strains causing human TB disease, and H37Ra) was assessed by multiple alignments using ClustalX. For Mtb32A, Mtb39A and Mtb72F, 15-mer human leukocyte antigen (HLA)-class II-binding peptides were predicted for 158 DRB1 alleles prevailing in populations with high TB burden, 6 DRB3/4/5, 8 DQ and 6 DP alleles, using NetMHCII-pan-3.0. Results for 3 DRB1 alleles were compared with previously published allele-matched *in vitro* binding data. Additional analyses were done for M72. Nonameric MHC class I-binding peptides in Mtb72F were predicted for three alleles representative of class I supertypes A02, A03 and B07, using seven prediction algorithms.

**Results:**

Sequence identity among strains was ≥98 % for each protein. Residue changes in Mtb39A comprised primarily single residue or nucleotide insertions and/or deletions in repeat regions, and were observed in 67 % of strains. For Mtb72F, 156 DRB1, 6 DRB3/4/5, 7 DQ and 5 DP alleles were predicted to contain at least one MHC class II-binding peptide, and class I-binding peptides were predicted for each HLA-A/B allele. Comparison of predicted MHC-II-binding peptides with experimental data indicated that the algorithm’s sensitivity and specificity were variable among alleles.

**Conclusions:**

The sequences from which Mtb72F and M72 are derived are highly conserved among representative *Mycobacterium* strains. Predicted putative T-cell epitopes in M72 and/or Mtb72F covered a wide array of HLA alleles. *In silico* binding predictions for class I- and II-binding putative epitopes can be complemented with biochemical verification of HLA binding capacity, processing and immunogenicity of the predicted peptides.

**Electronic supplementary material:**

The online version of this article (doi:10.1186/s12865-015-0119-7) contains supplementary material, which is available to authorized users.

## Background

Genetic variation in antigen components of vaccines at the pathogen level as well as in the host (the latter relating to the polymorphism in the human leukocyte antigen [HLA]), are major determinants for human vaccine efficacy. *Mycobacterium tuberculosis* (Mtb) is the causative agent of tuberculosis (TB) disease. Requisites for an efficacious TB vaccine to be used in populations world-wide are a minimal genomic variation of the target antigen among different Mtb strains, and the ability of the antigen’s contents of peptides to bind to the largest possible subset of HLA alleles in the host populations. The TB vaccine should also be able to induce robust T-cell responses [1]. While CD4^+^ T cells are thought to take center stage in the protection against Mtb infection, CD8^+^ T cells are also assumed to contribute to immunity against TB. Consequently, the selection of a vaccine antigen is in part focused on the abundance of HLA class I and class II-binding epitopes, which may be indicative of its immunogenicity.

HLA-peptide binding is a prerequisite for a putative epitope, linked to its correct processing and immunogenicity, and *in silico* prediction of this binding has become a tool in vaccine design. Predictive computer algorithms are generally trained by databases of experimentally validated epitopes with T-cell stimulation potential, such as SYFPEITHI [2] or the Immune Epitope Database (IEDB) [3]. For Mtb, the IEDB lists predominantly major histocompatibility complex class II (MHC-II)-restricted epitopes of the H37Rv strain [4], and the number of identified MHC class I (MHC-I)-restricted Mtb epitopes is limited [5]. Moreover, few Mtb epitopes have been identified for alleles prevailing in populations in TB-endemic regions. The known epitopes that are targeted by human T cell-mediated immune responses cover only 4 % of the Mtb proteome [6].

The *pe/ppe* gene family, which includes ~170 members, comprises approximately 10 % of the Mtb genome [7]. It has been suggested that proteins secreted by these genes aid in Mtb infection [8], and that Mtb39A (encoded by *Rv1196/ppe18)* has a role in Mtb virulence [9, 10]. Since infection can induce CD4^+^ and CD8^+^ T-cell responses to various PE/PPE proteins, some of these proteins have been evaluated as potential TB vaccine antigens [7, 11]. The vaccine antigen Mtb72F is a recombinant polyprotein derived from the H37Rv-expressed proteins Mtb39A and Mtb32A (the latter protein is a putative serine protease encoded by *pepA*/*Rv0125*) [12]. Both genes are present in both virulent and avirulent strains of the Mtb complex, and in bacille Calmette-Guérin (BCG) [13, 14]. The Mtb72F construct was generated by linking the carboxyl-terminal fragment of Mtb32A (Mtb32_C_), Mtb39A, and the amino-terminal fragment of Mtb32A excluding the signal sequence (Mtb32_N_) [12]. In a clinical study, the vaccine candidate Mtb72F induced comparable magnitudes of Mtb32A- and Mtb39A-specific CD4^+^ T-cell responses [15]. Collectively, at least 10 CD4^+^ or CD8^+^ T-cell epitopes (which were recognized by PBMC from human PPD-positive donors) have been identified in Mtb39A [14, 16], and a mouse CD8^+^ T-cell epitope was identified within Mtb32_C_ [12, 17].

Several Mtb72F peptides have been experimentally characterized to bind to one of three common HLA class II alleles (DRB1*01:01, 15:01 and 04:01) [18], or to the HLA class I allele B44 [16]. The successor of Mtb72F, M72, was generated from the Mtb72F sequence by introducing a point mutation in order to improve the long-term stability of the purified bulk of Mtb72F, and by modification of the N-terminal poly-histidine (poly-His) sequence. In clinical studies, these modifications were shown to have no effect on the antigen’s immunogenicity with respect to cell-mediated or humoral responses [19]. Indeed, antigen-specific CD4^+^ T-cell and antibody responses were elicited by AS02-adjuvanted Mtb72F vaccines in various adult populations [15, 19–21], and by AS01-adjuvanted M72 vaccines in populations of healthy adults in TB-endemic and non-endemic regions [19, 22, 23], HIV-1-infected adults in Switzerland [24], and healthy infants in The Gambia [25]. Consequently, M72 has been selected for further vaccine development.

The objectives of this *in silico* study were (1), to assess the sequence conservation of Mtb32A, Mtb39A, Mtb72F and M72, and (2), to predict MHC-I binding for nonameric peptides in Mtb72F, and MHC-II binding for 15-mer peptides in Mtb72F, Mtb32A and Mtb39A. The predictions focused on Mtb72F rather than M72 in order to allow comparison with previous prediction studies [26, 27], and additional evaluations were performed to assess the impact of the alterations introduced in Mtb72F to generate M72. MHC-II-binding predictions were evaluated for DRB1 alleles prevailing in populations with a high TB burden, and for DRB3/4/5, DP and DQ alleles. Last, we compared our prediction results obtained for three DRB1 alleles with allele-matched, experimentally derived MHC-II binding data described in [18].

Our findings suggested that the Mtb72F and M72 sequences are highly conserved among representative *Mycobacterium* strains and that the putative T-cell epitopes predicted for these two proteins cover a wide array of HLA class I and II alleles, while highlighting the limitations of *in silico* epitope predictions in general.

## Results

### Sequence conservation analysis for Mtb32A, Mtb39A, Mtb72F and M72

Using sequence similarity search programs, we assessed the similarity of H37Rv-derived Mtb32A and Mtb39A among genomes of *Mycobacterium* strains causing human TB disease, and H37Ra. For the 40 strains for which both sequences were available, similarity was also evaluated for the Mtb72F and M72 sequences constructed *in silico*. Furthermore, the presence of nucleotide and amino acid changes was evaluated for Mtb39A, given the relatively high frequency of sequence polymorphism reported for this protein [28, 29]. The strains evaluated included clinical isolates and representatives of all presently available Mtb strains, and comprised representatives of five of the six geographical lineages identified in a molecular phylogeny of the Mtb complex, which cover the predominant part of the Mtb geographical distribution [30].

For Mtb39A, we observed mainly single residue or nucleotide insertions and/or deletions with minor consequences for amino acid sequences, although amino acid changes were detected in 67 % of strains assessed (Table 1). Indeed, amino acid sequence comparisons revealed an average of 98 % identity for Mtb39A, with 100 % identity for 13 strains and at least 91 % identity among all strains excluding EAS054 and H37Ra. The latter strains contained one frameshift each, both associated with major amino acid changes of 117 and 62 residues, respectively, and consequently also with lower similarity (69 and 82 %, respectively). Relative to Mtb39A, sequence identity was higher for Mtb32A (*i.e.*, 99.6 % on average, and 100 % for 42 of the 44 strains assessed), consistent with earlier reports [28].

**Table 1:** Similarity of Mtb32A, Mtb39A, Mtb72F and M72 among selected *Mycobacterium* strains

Lineage^a^	Strains	NCBI Accession number	Mtb39A				Mtb39A	Mtb32A	Mtb72F	M72
			No. of aa changes	No. of aa/nt, position (pos)			% identity			
				Insertions	Deletions	Frameshifts				
1	T17	PRJNA55273	3				99.23	94.37	96.79	96.65
	T46	PRJNA55875	3				99.23^b^	-	-	-
	T92	PRJNA55099	3				99.23	100	99.58	99.44
	EAS054	PRJNA55133	117	1 nt, pos 811		1, pos 270-391	68.92	100	84.37	84.23
2	94_M4241A	PRJNA55095	0				100	100	100	99.86
	210 (Mtb Beijing*)*	PRJNA42617	30		1 aa, pos 274		91.3	100	95.4	95.26
	02_1987	PRJNA55097	1				99.74	100	99.86	99.72
	T85	PRJNA55131	1				99.74	100	99.86	99.72
	W-148 *(*Mtb*.* Beijing*;*MDR*)*	PRJNA182020	1				99.74	100	99.86	99.72
3	-						-	-	-	-
4	CDC1551	PRJNA57775	0				100	100	100	99.86
	C	PRJNA54359	0				100^b^	-	-	-
	F11	PRJNA58417	1				99.74	100	99.86	99.72
	GM1503	PRJNA55271	-	-	-	-	-	100^b^	-	-
	H37Ra^c^	PRJNA58853	62		1 nt, pos 922	1, pos 309–391	82.28	100	90.56	90.42
	Haarlem	PRJNA54453	0				100	100	100	99.86
	KZN1435 *(*MDR*)*	PRJNA59069	0				100	100	100	99.86
	98R604INHRIFEM	PRJNA55399	0				100	100	100	99.86
5	CPHL_A (*M. africanum)*	PRJNA55877	4		1 aa, pos 274		98.72	100	99.3	99.16
6	K85 (*M. africanum)*	PRJNA55879	3	2 aa, pos 162	1 aa, pos 274		98.48	100	99.17	99.03
n.d.	SUMu001	PRJNA51927	8		1 aa, pos 274		97.70	100	98.75	98.61
	SUMu002	PRJNA51925	-	-	-	-	-	100^b^	-	-
	SUMu003	PRJNA51931	2				99.49	100	99.72	99.58
	SUMu004	PRJNA51933	2		1 aa, pos 274		99.23	100	99.58	99.44
	SUMu005	PRJNA51935	2		1 aa, pos 274		99.23	100	99.58	99.44
	SUMu006	PRJNA91937	2				99.49	100	99.72	99.58
	SUMu008	PRJNA51941	-	-	-	-	-	100^b^	-	-
	SUMu010	PRJNA51945	-	-	-	-	-	100^b^	-	-
	KZN605 *(*XDR*)*	PRJNA54947	0				100	100	100	99.86
	KZN4207	PRJNA83619	0				100	100	100	99.86
	KZNR506 *(*XDR*)*	PRJNA47489	0				100	100	100	99.86
	KZNV2475 *(*MDR*)*	PRJNA47491	1				99.74	100	99.86	99.72
	UT205	PRJNA162183	0				100	100	100	99.86
	BTB05-552	PRJNA51871	0				100	100	100	99.86
	BTB05-559	PRJNA51873	0				100	100	100	99.86
	S96-129	PRJNA51869	0				100	100	100	99.86
	CCDC5079	PRJNA161943	1				99.74	100	99.86	99.72
	CCDC5180	PRJNA161941	1				99.74	100	99.86	99.72
	CTRI-2	PRJNA161997	0				100	100	100	99.86
	CTRI-4 *(*XDR*)*	PRJNA43175	3				99.23	100	99.58	99.44
	R1207 *(*MDR*)*	PRJNA46669	1				99.74	100	99.86	99.72
	X122 (pre*-*XDR*)*	PRJNA46667	1				99.74	100	99.86	99.72
	NA-A0008	PRJNA168604	6				98.47	100	99.58	99.44
	NA-A0009	PRJNA168605	3				99.23	100	99.16	99.02
	HN878	PRJNA46665	3				99.23	100	99.58	99.44
	RGTB423	PRJNA162179	2				99.23	85.63	92.48	92.34
	RGTB327	PRJNA157907	24	1 nt, pos 480	1 nt, pos 464	1, pos 139–155	93.91	100	96.67	96.53
				2 nt, pos 951	1 nt, pos 468	1, pos 314–316				
				1 nt, pos 959	1 nt, pos 469					
					1 nt, pos 470					
					1 nt, pos 475					
Affected Strains; no (%)			28 (67)	3 (7)	8 (19)	3 (7)				
Average % identity							98.08	99.55	98.71	98.57

^a^Lineages as defined in ref. [30]: no. 1: The Philippines / rim of the Indian Ocean; no. 2: East Asia; no. 3: India / East Africa; no. 4: Europe / Americas; no. 5 and no. 6: West African 1 and 2, respectively. n.d., not defined. MDR*,* multiple drug-resistant; (pre-)XDR, (pre-)extensively drug-resistant. aa, amino acid(s); nt, nucleotide(s). ^b^Evaluations not performed for Mtb72F/M72 as a complete sequence was only available for one of the two genes. % identity = (number of identical residues / length of alignment) x100, as calculated from multiple alignment comparisons. Mtb39A genome sequences were derived from complete genomes (chromosomes; highlighted in bold) or assembled partial genomes (scaffolds/contigs; normal font). ^c^Laboratory strain H37Ra was used as reference

Mtb32A and Mtb39A sequences in 13 clinical isolates were identical to those in Mtb72F, and 84–99.9 % sequence identity was observed for the remaining strains. Among the three extensively drug-resistant (XDR), 1 pre-XDR and four multiple drug-resistant (MDR) strains assessed, percentages identity for Mtb72F were at least 99.6 %. Overall, percentages identity were 0.14 % (1/718) lower for M72 than for Mtb72F due to the introduced point mutation in M72, and exceeded 98 % for both antigens in at least one strain per selected Mtb lineage.

### MHC-II-binding peptides in Mtb32A, Mtb39A and Mtb72F

A computerized algorithm (NetMHCIIpan-3.0) was used to compute MHC-II binding predictions for 15-mer peptides in Mtb72F and (H37Rv) Mtb32A and Mtb39A, for 158 HLA-DRB1, 6 DRB3/4/5, 8 DQ and 6 DP alleles. The DRB1 alleles were selected based on their presence in populations in four regions with a high TB prevalence (Northern, Southern and North-Eastern India and China) and in the native population of Sub-Saharan Africa, which has a high TB incidence. The 6 most common DRB3/4/5 alleles [31] and the subset of DQ/DP molecules that are present in at least 85 % of populations worldwide [32, 33] were also included, and together our selection contained the full panel of 46 DR, DP and DQ alleles reported to collectively cover almost 90 % of populations worldwide [31].

**Fig. 1:**
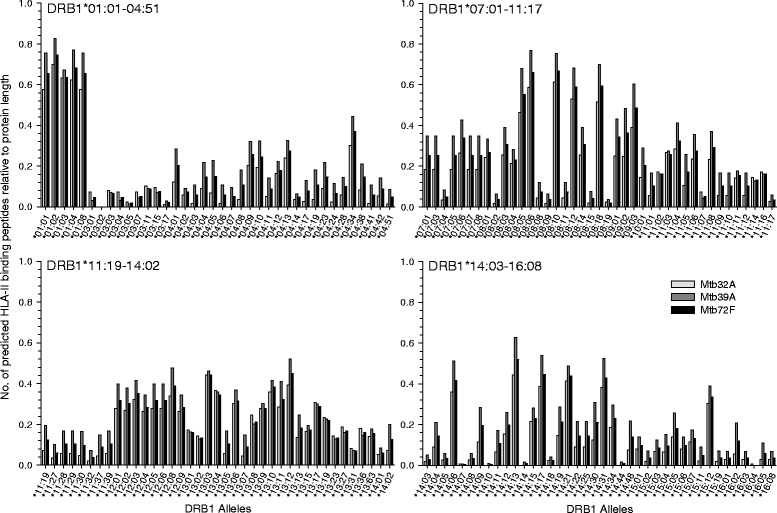
Numbers of predicted HLA class II-binding peptides for Mtb32A, Mtb39A and Mtb72F. HLA-II binding predictions were generated for 15-mer peptides (overlapping by 14 amino acids) of Mtb32A, Mtb39A and Mtb72F using NetMHCIIpan-3.0, for 158 DRB1 alleles. Results were normalized with respect to the different protein lengths of Mtb32A, Mtb39A and Mtb72F (*i.e.*, 355, 391 and 729 amino acids, respectively)

HLA-II binding peptides predicted for Mtb72F were found to cover a wide array of alleles (Additional file 1). For DRB1*15 and DRB1*16, which have been linked to susceptibility to TB in Indian populations [34, 35], we predicted an average of 64 binding peptides in Mtb72F per allele for the three defined Indian regions. Across geographical regions, the highest numbers of binding peptides for Mtb72F were predicted for DRB1*01:02, 01:04, 08:10, 08:06, 01:01 and 01:08, *i.e.*, 533–468 peptides/allele, or 0.65-0.75/allele after normalization of the results with respect to the different protein lengths of Mtb32A, Mtb39A and Mtb72F (Fig. 1). Average numbers of predicted binding peptides per allele in Mtb72F were highest in Sub-Saharan Africa (163 per allele) followed by the individual Indian regions (115–143 per allele) and China (131 per allele) (Table 2). There was a trend for higher numbers of binding peptides for Mtb39A relative to Mtb32A.

**Table 2:** MHC class II-binding peptide predictions for common DRB1 alleles in high-TB burden regions

Region/Alleles assessed (N)	Protein	Predicted epitopes (N)		DRB1 alleles without predicted epitope	
		Total	Average/allele	N	Allele
*Overall*	Mtb32A	8792	56	5	*03:02, *04:07, *14:10, *14:14, *14:44
(*N* = 158)	Mtb39A	14675	93	2	*03:02, *16:04
	Mtb72F	22065	140	2	*03:02, *16:04^a^
*China*	Mtb32A	5873	40	5	*03:02, *04:07, *14:10, *14:14, *14:44
(*N* = 146)	Mtb39A	12794	88	2	*03:02, *16:04
	Mtb72F	19109	131	2	*03:02, *16:04^a^
*N. India*	Mtb32A	2595	55	2	*03:02, *04:07
(*N* = 47)	Mtb39A	4516	96	1	*03:02
	Mtb72F	6733	143	1	*03:02^a^
*S. India*	Mtb32A	1087	43	0	
(*N* = 25)	Mtb39A	1993	80	1	*16:04
	Mtb72F	2868	115	1	*16:04^a^
*N.E. India*	Mtb32A	2155	51	2	*03:02, *04:07
(*N* = 42)	Mtb39A	3517	84	1	*03:02
	Mtb72F	5298	126	1	*03:02^a^
*India (Total)*	Mtb32A	2807	37	2	*03:02, *04:07
(*N* = 76)	Mtb39A	4634	61	2	*03:02, *16:04
	Mtb72F	6876	90	2	*03:02, *16:04^a^
*S.S. Africa* ^b^	Mtb32A	4447	67	1	*03:02
(*N* = 66)	Mtb39A	6977	106	1	*03:02
	Mtb72F	10737	163	1	*03:02^a^

Prediction results represent the sum of the weak and strong binders. ^a^Allele frequencies in the populations assessed are included in Additional file 2. ^b^S.S. Africa, Sub-Saharan Africa, native population only

Across regions, no peptides were predicted to bind to five alleles (DRB1*03:02, 04:07, 14:10, 14:14 and 14:44) for Mtb32A, and to two alleles (DRB1*16:04 and 03:02) for Mtb39A. Consequently, binding peptides were also not predicted for DRB1*16:04 and 03:02 for Mtb72F, since for DRB1*16:04 all binding peptides were predicted to be located in the Mtb32A signal sequence (which is absent in Mtb72F), while for DRB1*03:02 no binding peptides were predicted for any part of Mtb32A or Mtb39A. Reported allele frequencies in certain Sub-Saharan African, Chinese and Indian populations (as extracted from a public database [36]) are relatively low for DRB1*16:04 (ranging from 0.0-0.6 %) and higher for DRB1*03:02 (ranging from 0.0–17.2 %; Additional file 2).

For the 6 DRB3/4/5, 8 HLA-DQ and 6 HLA-DP molecules, we predicted up to 145, 614 and 170 binding peptides in Mtb72F per allele, respectively (Table 3). Binding peptides in Mtb72F were predicted for each DRB3/4/5 and DP/DQ allele assessed, with the exception of DQA1*02:01-DQB1*02:01 (for which there were no prediction results for any of the four proteins assessed) and DPA1*02:02-DPB1*05:01 (for which all predicted peptides were located in the Mtb32A signal sequence). Worldwide, genotypic frequencies of DQA1*02:01-DQB1*02:01 and DPA1*02:02-DPB1*05:01 are 6 % and 12 %, respectively [37], but for the populations assessed, no frequencies of these alleles have been reported in the used database (Additional file 2). For DQA1*02:01-DQB1*02:01 in haplotype with DRB1*07:01 (either alone or with DPB1*02:01, 03:01 or 17:01) frequencies in various populations ranged from 1–15 %. For DRB1*07:01, and thus also for the DRB1*07:01-DQA1*02:01-DQB1*02:01 haplotypes, 181 peptides were predicted in Mtb72F (Additional file 1), with potentially higher numbers for haplotypes that also include DPB1*02:01 (Table 3).

**Table 3:** Prediction of HLA II-binding peptides for DRB3/4/5, DP and DQ alleles

HLA molecule	Mtb32A	Mtb39A	Mtb72F	M72
DRB3*01:01	18	7	31	31
DRB3*02:02	19	56	71	71
DRB3*03:01	51	82	125	125
DRB4*01:01	27	38	58	58
DRB5*01:01	32	85	109	109
DRB5*01:02	45	107	145	145
DQA1*05:01-DQB1*02:01^a^	14	35	51	51
DQA1*02:01-DQB1*02:01^b^	0	0	0	0
DQA1*05:01-DQB1*03:01^a^	291	325	614	610
DQA1*03:01-DQB1*03:02^a^	17	75	88	88
DQA1*04:01-DQB1*04:02^a^	15	75	87	88
DQA1*01:01-DQB1*05:01^a^	8	0	1	1
DQA1*01:02-DQB1*05:02	26	37	61	61
DQA1*01:02-DQB1*06:02^a^	161	262	392	392
DPA1*02:01-DPB1*01:01^c^	27	33	56	56
DPA1*01:03-DPB1*02:01^c^	20	23	39	39
DPA1*01:03-DPB1*04:01^c^	14	11	16	16
DPA1*01:03-DPB1*04:02^c^	18	17	28	28
DPA1*02:02-DPB1*05:01^b, c^	8	0	0	0
DPA1*02:01-DPB1*14:01	50	128	170	170

Prediction results represent the sum of the weak and strong binders. ^a^ Allele is among the 6 HLA-DQ molecules that reportedly are present in >85 % of populations worldwide [32]. ^b^ For these alleles, no frequencies are reported in the populations assessed (Additional file 2). ^c^ Allele is among the 5 HLA-DP molecules that reportedly are present in >90 % of populations worldwide [33]

### Impact of the alterations made to construct Mtb72F and M72, on MHC-II binding predictions

Several alterations have been introduced into the native Mtb32A sequence in order to construct Mtb72F [12]; summarized in Table 4), and hence we have assessed the potential impact thereof on the prediction results for Mtb72F. We observed that the alterations resulted in a reduction of 42 predicted binding peptides, as well as in 1 and 18 additional predicted (non-Mtb) binding peptides owing to the addition of the poly-His tag and the hinge sequences respectively. As described in the previous section, no binding peptides were predicted after the alterations for one allele (DRB1*16:04) due to the deletion of the Mtb32A signal sequence. Additional evaluations were performed to assess the impact of the modifications introduced into Mtb72F in order to generate M72, comprising the change of the N-terminal poly-His sequence from MHHHHHH to MHH, and the substitution of one serine residue for an alanine residue. Neither of the modifications resulted in a change in the number of alleles with at least one predicted putative epitope. No putative epitopes were predicted to be present in the MHH tag, and thus this modification of the poly-His tag resulted in the loss of the above described non-Mtb binding peptide predicted for the Mtb72F poly-His tag.

**Table 4:** Impact of the alterations introduced for the Mtb72F construction on the peptide binding predictions

Alteration introduced in Mtb72F	Change in the numbers of covered^a^ alleles for Mtb72F	Explanation
Deletion of the Mtb32A signal sequence.	Loss of 1 covered allele containing 28 predicted epitopes	Epitopes in the Mtb32A signal sequence were predicted for 149 of the 158 alleles assessed. For 148 of the 149 alleles, epitopes were also predicted for the other parts of the protein. Only for 1 allele (DRB1^*^16:04), all 28 predicted epitopes were located in the Mtb32A signal sequence, and were thus not predicted for Mtb72F.
Splitting the Mtb32A sequence upstream and downstream of ‘TAAS’ sequence.	No changes in the number of covered alleles.	For each allele with an epitope predicted in this part of the protein there was also an epitope predicted in other parts of the protein. There was an overall loss of 14 predicted epitopes.
Addition of a poly-His tag (MHHHHHH) at the Mtb32A C-terminal end.	No changes in the number of covered alleles.	One epitope (MHHHHHHTAASDNFQ, binding to DRB1^*^08:18) was predicted for the Meth-His tag in Mtb72F. There were also other epitopes predicted for this allele.
Addition of 2-amino acid hinge sequences at the junction sites between Mtb32_C_ and Mtb39A (EF), and between Mtb39A and Mtb32_N_ (DI).	No changes in the number of covered alleles.	Adding the EF and DI sequences resulted in 13 and 5 additional predicted epitopes, binding to 43 and 31 alleles, respectively. However, the number of alleles with at least one predicted epitope did not change.

^a^ Covered allele: an allele for which at least one epitope was predicted

### Comparison of MHC-II binding predictions for 3 DRB1 alleles with experimental data

We compared our prediction results for three common HLA class II alleles (DRB1*01:01, 04:01 and 15:01) with binding data obtained *in vitro* for Mtb32A and Mtb39A from Maeurer et al. [18]. In the latter study, the binding to soluble recombinant HLA-II monomers of three alleles was experimentally determined for 7466 15-mer Mtb peptides.

For Mtb72F predictions, the algorithm’s sensitivity was higher for DRB1*01:01 than for DRB1*04:01 or DRB1*15:01, as 74, 28 and 33 % respectively of the allele-matched experimentally identified binding peptides were also predicted by the algorithm, while the reverse was found for the specificities (which were 34, 81 and 90 %, respectively; Table 5). The same trends were observed for the prediction results obtained for Mtb32A and Mtb39A separately.

**Table 5:** Comparison of experimentally-defined and predicted 15-mer HLA class II-binding peptides for three DRB1 alleles

Performance algorithm	DRB1*0101 (DR1)			DRB1*1501 (DR2)			DRB1*0401 (DR4)		
	**Mtb32A**	**Mtb39A**	**Mtb72F**	**Mtb32A**	**Mtb39A**	**Mtb72F**	**Mtb32A**	**Mtb39A**	**Mtb72F**
	**INAFSVGSGQTYGVD**	VNEAEYGEMWAQDAA		LSQDRFADFPALPLD	**MLKGFAPAAAAQAVQ**		**LNGLIQFDAAIQPGD**	**TAYGLTVPPPVIAEN**	
	GSGQTYGVDVVGYDR	**AYETAYGLTVPPPVI**		DRFADFPALPLDPSA	KTVSPHRSPISNMVS		NFQLSQGGQGFAIPI	VVWGLTVGSWIGSSA	
	**ATDINAFSVGSGQTY**	**AEYGEMWAQDAAAMF**		APAQAAPPALSQDRF^**a**^	MSSLGSSLGSSGLGG		LNGHHPGDVISVTWQ	GLTVGSWIGSSAGLM	
	**YDRTQDVAVLQLRGA**	**GEMWAQDAAAMFGYA**			**SSAGLMVAAASPYVA**		LTNNHVIAGATDINA	GVAANLGRAASVGSL	
	**VAVLQLRGAGGLPSA**	**AAAAYETAYGLTVPP**					QTYGVDVVGYDRTQD	ANLGRAASVGSLSVP	
	GGQGGTPRAVPGRVV	**ASVGSLSVPQAWAAA**					VPGRVVALGQTVQAS	AAAAYETAYGLTVPP	
	QTYGVDVVGYDRTQD	**VVWGLTVGSWIGSSA**					IPIGQAMAIAGQIRS	ASAFQSVVWGLTVGS	
	FSVGSGQTYGVDVVG	**VRVAAAAYETAYGLT**					NGARVQRVVGSAPAA	VTPAARALPLTSLTS	
	**IPIGQAMAIAGQIRS**	AIAVNEAEYGEMWAQ					INAFSVGSGQTYGVD	**LMILIATNLLGQNTP**	
		**MLKGFAPAAAAQAVQ**					**RVQRVVGSAPAASLG**	VAAASPYVAWMSVTA	
		**AENRAELMILIATNL**					AGQIRSGGGSPTVHI	**ASPYVAWMSVTAGQA**	
		**ASAFQSVVWGLTVGS**					GSGQTYGVDVVGYDR	**VGSWIGSSAGLMVAA**	
		**FSAASAFQSVVWGLT**					**LQLRGAGGLPSAAIG**	AENRAELMILIATNL	
		**SSAGLMVAAASPYVA**					IAGATDINAFSVGSG	SASLVAAAQMWDSVA	
							RVVALGQTVQASDSL	**SSAGLMVAAASPYVA**	
							YDRTQDVAVLQLRGA	LPPEINSARMYAGPG	
							FSVGSGQTYGVDVVG	GQAELTAAQVRVAAA	
							RVVGSAPAASLGIST	**MLKGFAPAAAAQAVQ**	
							**VAVLQLRGAGGLPSA**	**AAQVRVAAAAYETAY**	
							GFAIPIGQAMAIAGQ	**YVAWMSVTAGQAELT**	
							GVDVVGYDRTQDVAV	**DAAAMFGYAAATATA**	
							ATDINAFSVGSGQTY	PSSKLGGLWKTVSPH	
							PLDPSAMVAQVGPQV	VTAGQAELTAAQVRV	
							TQDVAVLQLRGAGGL	AVQTAAQNGVRAMSS	
							GGTPRAVPGRVVALG	**LIATNLLGQNTPAIA**	
							NHVIAGATDINAFSV	**GEMWAQDAAAMFGYA**	
							GGGSPTVHIGPTAFL	MYAGPGSASLVAAAQ	
							GTGIVIDPNGVVLTN	VRVAAAAYETAYGLT	
							RWSWLLSVLAAVGLG^**a**^	**FSAASAFQSVVWGLT**	
								AIAVNEAEYGEMWAQ	
								**AYETAYGLTVPPPVI**	
								ASVGSLSVPQAWAAA	
Total binders determined *in vitro*	9	14	23	3	4	6	29	32	60
Total non-binders^b^	106	113	202	112	123	219	86	95	165
True predicted binders (TP)	5	12	17	0	2	2	4	13	17
True predicted non-binders (TN)	45	28	68	102	108	197	77	70	134
**Sensitivity prediction algorithm**	**55 %**	**86 %**	**74 %**	**0 %**	**50 %**	**33 %**	**14 %**	**41 %**	**28 %**
**Specificity prediction algorithm**	**42 %**	**25 %**	**34 %**	**91 %**	**88 %**	**90 %**	**90 %**	**74 %**	**81 %**

Listed epitopes were derived from ref. [18] and experimentally found to bind MCH Class II molecules. Peptides predicted by NetMHCpan-3.0 in the current study are underlined and highlighted in bold. *TP* True positives. In ref. [18] and the current study, data were generated using 15 mer peptides overlapping by 12 mer. *TN* True negatives. ^a^Sequence does not appear in the Mtb72F recombinant protein. ^b^Length of the Mtb72F sequence is 729 amino acids, containing 225 overlapping15-mer peptides. Mtb32A and Mtb39A contain 115 and 127 overlapping 15-mer peptides

### MHC-I binding predictions for nonameric peptides in Mtb72F

HLA I-binding 9-mer peptides in Mtb72F were predicted for the A*02:01, A*03:01 and B*07:02 alleles (representing the HLA-I supertypes A02, A03 and B07 respectively [38]). Collectively these supertypes have been reported to cover 80–90 % of any given human population worldwide, regardless of ethnicity [39].

Using NetMHCpan-2.2, the combined numbers of strong and weak binders predicted for A*02:01, A*03:01 and B*07:02 were 23, 2 and 15, respectively (Additional file 3). The 11 strong binders predicted by this algorithm, and the 10 high-affinity binders predicted by NetCTLpan-1 were all restricted to A*02:01 or B*07:02. Higher numbers of binding peptides (78 in total, of which 48 strong binders) were predicted by the combined 5 other algorithms that were used.

## Discussion

This *in silico* study explored the sequence conservation of Mtb32A, Mtb39A, Mtb72F and the current candidate vaccine antigen M72, and predicted MHC-I and/or MHC-II-binding peptides for these proteins. Our results led to two main conclusions. First, the Mtb32A and Mtb39A proteins (and thus also the Mtb72F and M72 constructs) appeared to be well conserved (with at least 98 % identity) among the strains representative for the major part of the Mtb geographical distribution (including MDR, XDR and pre-XDR strains), with for Mtb39A a higher sequence conservation than previously reported in [26, 27]. Second, the putative CD4^+^ T-cell epitopes predicted for Mtb72F and M72 were shown to cover a broad range of HLA Class II DRB1, DRB3/4/5, DQ and DP alleles, and the putative CD8^+^ T-cell epitopes predicted for Mtb72F covered each of the HLA-A or -B alleles assessed.

Hyperconservation has been reported for the large majority of human T-cell epitopes in the Mtb complex, although *pe/ppe* genes were excluded from that study [40]. Previously, *mtb32a* sequences of H37Ra, H37Rv, Erdman and the clinical isolate CSU93 were found to be identical [13], and, extending these studies, Mtb32A was shown to be relatively well conserved among several clinical isolates, with amino acid changes in only 6 % of the 225 investigated strains [28]. Consistently, our data revealed a high level of conservation for Mtb32A. For Mtb39A, our findings contrast with previous studies which suggested a substantial genetic variation for this protein, potentially associated with the homologous recombination with *ppe19* and *ppe60* [28, 29]. Nonetheless, several of the minor insertions and/or deletions we observed in the Mtb39A sequence (the 2-aa insertion at the 162nd base and the 1-aa deletions at the 274th base, in repeat regions in 1 and 6 strains, respectively) may be identical to those reported for Arkansas-derived samples in the above study [28]. Of the frameshifts leading to major amino acid changes, as we observed in 3 strains, the one in the avirulent H37Ra strain was consistent with earlier findings [41] and of little clinical relevance, while the one in EAS054 has not been reported previously to the best of our knowledge. Possibly, the number and origin of the strains evaluated here reflect the worldwide prevalence of Mtb more accurately than those used in the referenced studies, which included 225 US and Turkey-derived clinical strains [28], or 16 clinical isolate sequences from a public database [29]. As we have not assessed whether the minor insertions and/or deletions coincide with locations of predicted putative epitopes, the impact of the associated residue changes on the recognition of clinical Mtb strains by T-cell responses induced by Mtb72F or M72 candidate vaccines, is not known.

MHC-II binding peptides in Mtb72F were predicted to cover all DRB1, DRB3/4/5, DQ and DP alleles assessed, except for 2 DRB1 alleles (03:02 and 16:04), 1 DQ and 1 DP allele. Based on the used database, this DP allele (DPA1*02:02-DPB1*05:01) does not occur in the populations assessed, while the DQ allele (DQA1*02:01-DQB1*02:01) only prevails in these populations in haplotype with DRB1*07:01, which was predicted to be well covered with binding peptides. Several aspects are important to consider with respect to the implications of the absence of predicted binding peptides for the two DRB1 alleles for the candidate vaccines’ potential efficacy in the populations concerned. First, except for the higher frequency of DRB1*03:02 in certain Sub-Saharan African populations (≤17.2 %) as compared with the worldwide genotypic frequency of 1.1 % [37], the associated allele frequencies were generally low in these populations (≤1.5 %). Moreover, a potential absence of binding peptides for the two DRB1 alleles in these populations may be compensated by peptides present on other HLA-DR, −DP or -DQ alleles, and vice versa. For instance, for the South-African Limpopo Venda population, DRB1*13:02 and DQB1*03:01–03:04 have been associated with TB disease [42]. In this population, the frequency of DRB1*03:02 is relatively high (9 %). The absence of binding peptides for DRB1*03:02 may in this case be compensated by several other alleles prevailing at high frequencies in this population (e.g., DRB1*11, DRB1*03:01, DRB1*13:01, DRB*01 and DQB1*06 and DQB1*05; data retrieved from [36]), for which we predicted high numbers of binding peptides per allele in Mtb72F. It may also be reassuring that in a recent clinical trial, the candidate vaccine M72/AS01 was able to induce robust CD4^+^ T-cell responses in healthy adults in South Africa [22]. Furthermore, it is noted that for other antigens (meningococcal serogroup B proteins) it was shown that a set of only 2 predicted T-cell epitopes could theoretically cover large proportions of over 11 populations worldwide [43]. A confounding factor is that the allele frequencies sourced from a public database may have only partial or local coverage (e.g. cities or ethnic groups), since they are usually based on relatively small sample sizes rather than population statistics. Similarly, since certain alleles specific to susceptible populations have likely been the prime focus of several studies, the database may contain disproportionally high numbers of such alleles.

Although our data showed that the changes introduced in Mtb32A in order to construct Mtb72F may result in an overall reduction of Mtb binding peptides, only two alleles present in the populations assessed (DRB1*03:02 and 16:04) were predicted to have no binding peptide in Mtb72F. The other modifications (addition of hinge sequences and a poly-His tag) were predicted to jointly generate 19 binding peptides, of which one peptide was predicted to be absent in M72. Since non-Mtb epitopes do not contribute to the vaccine’s ability to induce Mtb-specific responses, their relatively low frequency in Mtb72F and M72 is reassuring.

Previously, *in silico* MHC-II binding predictions for 9-mer peptides in Mtb72F have been generated for 34 DRB1 alleles [26, 27]. Comparison of the previous data with the present predictions for 15-mer peptides and DRB1 alleles revealed up to 37-fold differences in the allele-matched binding peptide numbers, which can likely for a great part be explained by the different binding peptide lengths assumed by the prediction algorithm. HLA-I and HLA-II binding peptides are composed of 8–10 and 13–17 amino acids respectively [44]. The peptide-binding groove of MCH-II molecules is open-ended, which complicates identification of the 9-mer core peptides and the flanking residues (which may also contain predictive information [45]). MHC-II prediction algorithms assuming 9-mer binding peptides do not use information present outside the core, leading to lower accuracy [46]. Consistently, it was shown that including flanking residues among inputs improved the performance of MHC-II prediction methods [47, 48]. However, even predictions generated by algorithms assuming 15-mer binding peptides may have limited reproducibility, as suggested by the present results. Indeed, comparison of our results for Mtb32A and Mtb39A with allele-matched experimental binding data [18] revealed that the algorithm’s sensitivity and specificity were variable and occasionally rather low for Mtb72F (e.g., 28 %). It is noted though that due to the lack of additional suitable *in vitro* data, this comparison covered only 3 of the 158 HLA-DRB1 alleles, and none of the DRB3/4/5, DQ and DP alleles assessed in our study. While the observed variability in the performance of the algorithm may be a consequence of the limited extent of this evaluation, it could therefore also be inherent to the limitations of the used prediction algorithms for peptide binding in general. Similarly, most algorithms predicting T-cell activation for class II binding peptides display low sensitivity, as less than half of the defined T-cell activating peptides were predicted to be binders [46]. Thus, epitope identification requires an integrated approach, in which *in silico* binding predictions are complemented with confirmatory biochemical verification of MHC processing and binding, and of the ability of the predicted peptides to induce T-cell responses [49]. Other useful tools are *in silico* methods that enable linking binding affinity patterns to topological characteristics [50].

Due to the capped MHC-I peptide binding groove, this binding is more specific than binding to MHC-II, and HLA-I binding prediction algorithms are considered relatively accurate [46, 49]. Indeed, there was only at most a five-fold difference between the previously reported numbers of HLA A- and B-binding peptides in Mtb72F [27] and the numbers per allele computed here.

In the present study, the majority of predicted peptides were presented by A*02:01, listed in the IEDB among the most frequent HLA class I alleles in regions with high TB incidence [4]. The predicted high affinity-binders were mainly presented by A*02:01 and B*07:02 rather than A*03:01, consistent with data obtained for a panel of 432 H37Rv-derived peptides [5], while other Mtb proteins (e.g. Mtb8.4, CFP10 and Ag85B) were shown, experimentally or *in silico*, to be primarily presented by various HLA-B molecules [51, 52]. Furthermore, our data showing the predicted binding of the Mtb72F peptides APINSATAM, APAAAAQAV, LLGQNTPAI and SSKLGGLWK to B*07:02, B*07:02, A*02:01 and A*03:01 respectively corroborates earlier predictions for these peptides [5]. Of note, several previously predicted HLA I-restricted Mtb peptides were experimentally shown to induce CD4^+^ rather than CD8^+^ T cell responses [53]. Similarly, the HLA-B44-restricted epitope MWAQDAAAMF [16], was also found in two 15-mer Mtb39A peptides that were experimentally defined [18] and predicted here to bind to DRB1*04:01 and/or 01:01.

Last, for cytomegalovirus antigens, it has been suggested that less than 20 uniquely defined HLA-restricted CD8^+^ T-cell epitopes could provide 90 % coverage of the three major ethnic groups [54]. Possibly, the putative class I Mtb epitopes predicted for Mtb72F could also have a wide coverage of populations worldwide, and, given that only minor changes were introduced into Mtb72F to generate M72, this would also apply to M72.

## Conclusions

Mtb72F and M72 sequences were shown to be highly conserved among *Mycobacterium* strains including MDR and XDR strains, and are thus expected to provide broad coverage of the pathogen population. For Mtb72F, the predicted MHC-II binding peptides covered a wide array of HLA-DRB1, −DQ and -DP alleles, whereby the absence of predicted binding peptides for 2 DRB1 alleles prevailing in some populations with high TB burden may be compensated by peptides binding to other HLA loci. MHC-I binding peptides were predicted for each HLA-A/B allele assessed. Yet, discrepancies between the current predictions and experimental HLA-binding data underscore the limitations inherent in interpreting *in silico* predictions for HLA class II-restricted peptides. Experimental epitope verification, by determination of the HLA-binding capacity of synthetic peptides and of their correct processing, remains valuable. Also, their immunogenicity could be confirmed by identification of T cells specific for the epitope, using PBMCs from HLA-typed subjects.

## Methods

### Mtb72F and M72 vaccine antigens

Mtb72F is a 729-aa, 72-kDa polyprotein derived from Mtb32A and Mtb39A (encoded by *Rv0125* and *Rv1196*, respectively). The full nucleotide and deduced amino acid sequences of Mtb32A, Mtb39A and Mtb72F have been described [12–14]. Mtb72F has been constructed in the linear order (starting at the amino end): carboxyl-terminal portion of Mtb32A (Mtb32_C_) - full-length ORF of Mtb39A - N-terminal fragment of Mtb32A excluding the signal sequence (Mtb32_N_). Since the Mtb32A sequence has been split upstream and downstream of the TAAS sequence linking the Mtb32_C_ and Mtb32_N_ sequences, TAAS is present in Mtb72F at the N-terminal end of Mtb32_C_ as well as at the C-terminal end of Mtb32_N_. In summary, the following changes were made compared to the native Mtb32A protein: (i) addition of nucleotide sequences encoding a poly–His tag (MHHHHHH) at the Mtb72F N-terminus; (ii) deletion of the 32-aa signal sequence (MSNSRRRSLRWSWLLSVLAAVGLGLATAPAQA) located at the Mtb32 N-terminus; (iii) addition of two hinge sequences (an *Eco*RI restriction site, located between Mtb32_C_ and Mtb39A, and an *Eco*RV restriction site between Mtb39A and Mtb32_N_) resulting in the EF and DI residues in Mtb72F, respectively (one at each junction site).

M72 was generated by introducing a point mutation in Mtb72F, thereby replacing Ser_706_ by Ala_706_, and by changing the poly-His tag of Mtb72F into the MHH sequence present in M72.

### Sequence alignments and similarity analyses

Using the BLAST program [55], Mtb protein sequences available in the NCBI GenPept database (accessed February 2013) were screened for the presence of Mtb32A and Mtb39A sequence. The selection of 44 and 42 genomes for Mtb32A and Mtb39A respectively comprised the laboratory strain H37Ra, as well as *M. africanum* and clinical isolates from presently available Mtb strains including MDR, XDR and pre-XDR strains (*i.e.*, Mtb Beijing family strains and North and South-American, African, Asian, and European strains). To the extent the information was available, the strains were matched with a detailed molecular phylogeny of the Mtb complex [30], which divides the global population structure of Mtb strains in 6 main phylogeographical lineages. Our selection included predominant strains from each main lineage, with the exception of Lineage 3 (India/East Africa) for which no strains were included (Table 1).

Complete genomes, or assembled partial genomes when validated and/or complete genomes were unavailable, were downloaded from the GenBank database. For the 40 strains for which both the Mtb32A and Mtb39A sequences were available, the genomes were used for the construction of Mtb72F and M72 as described above (see ‘*Mtb72F and M72 vaccine antigens’*), excluding the additional residues at the junction sites and the Mtb72F/M72 poly-His tag). To ensure that the used sequence was specific for *mtb39*, rather than for one of the known *mtb39* paralogs (*ppe19* and *ppe60*), a fragment containing Mtb39A with PE13 upstream, and ESAT-6-like (*esxL*) downstream, was extracted from the TB genomes. Mtb39A nucleotide sequences from 42 strains were translated into amino acid sequences, which were subsequently compared by multiple alignments using ClustalX software. For Mtb72F and M72, all protein sequences had very similar or identical lengths. Similarity analyses for Mtb32A, Mtb39A, Mtb72F and M72 were done by multiple alignment comparisons. Percentage identity between the sequences was defined as ‘(number of identical residues / length of alignment) x 100’. The data for Mtb39A were also analysed separately for the presence of nucleotide and amino acid changes (insertions, deletions and/or frameshifts).

### DRB, DP and DQ allele frequencies

MHC-II peptide binding predictions were done for HLA-DRB1, DRB3/4/5, DQ and DP alleles, whereby the DRB1 allele frequencies characterizing the populations in each region were extracted from the ‘Allele*frequencies in Worldwide Populations’ database [36] (http://www.allelefrequencies.net; accessed November 2013). The 158 DRB1 alleles evaluated (including the common alleles 01:01, 03:01, 04:01, 07:01, 11:01 and 15:01) comprised 146, 47, 25, 42 and 66 alleles present in populations in China, North India, Southern India, North-Eastern India and Sub-Saharan Africa (native population), respectively. India and China, and 9 Sub-Saharan African countries are among the 22 high-TB burden regions defined by the World Health Organization [56]. The discrimination of Northern, Southern, and North-Eastern Indian regions was based on the local prevalent population structures, while for the Chinese and Sub-Saharan African regions insufficient information was available to apply further detailing. The 8 HLA-DQ molecules assessed contained the 6 molecules that reportedly are present in over 85 % of populations worldwide [32], and the 6 HLA-DP molecules assessed contained the 5 HLA-DP molecules that reportedly are present in >90 % of populations worldwide [33] (see Table 3). Frequencies of alleles for which no binding peptides were predicted in this study were extracted from the above-mentioned public database [36]; accessed 26 January 2015). HLA-I binding predictions were done for A*02:01, A*03:01 and B*07:02, which were selected by the developers of the NetMHCpan-2.2 algorithm to represent the supertypes A02, A03 and B07 respectively [38, 57]. Collectively these supertypes have been reported to cover 83–88 % of 5 major ethnic groups worldwide [39].

### HLA class II-binding peptide predictions for DRB, DP and DQ alleles

HLA-II binding predictions were generated for 15-mer peptides (overlapping by 14 amino acids) of Mtb32A, Mtb39A and Mtb72F using NetMHCIIpan-3.0 [58], and for the 158 DRB1, 6 DRB3/4/5, 6 DP and 8 DQ alleles described. The Mtb72F construct was produced *in silico* as described above (see ‘*Mtb72F and M72 vaccine antigens**’*) with exclusion of the poly-His sequence. In addition, the potential impact of the alterations introduced for the Mtb72F and M72 constructions on the prediction results was evaluated. For DRB1*01:01, DRB1*04:01 and DRB1*15:01, data were also generated using 15 mer peptides overlapping by 12 amino acids. Results were compared with previously published experimental data for 15-mer Mtb peptides (overlapping by 12 amino acids) binding to these 3 alleles [18], using the Mtb72F sequence with exclusion of the hinge sequences and poly-His tag. As recommended by the program, a peptide was identified as a ‘strong binder’ if the binding affinity [IC_50_] was below 50 nM or as a ‘weak binder’ if the IC_50_ was >50 nM but <500 nM. The total numbers of epitopes shown in this study represent the sum of the strong and the weak binders.

### HLA class I-binding peptide predictions

MHC-I-binding predictions for the A*02:01, A*03:01 and B*07:02 alleles were done for 9-mer peptides in Mtb72F using NetCTLpan-1 [59], NetMHCpan-2.2 [57], SVMHC, Syfpeithi [2], EpiJen [60], nHLA-pred (nHLA-compred and nHLA-aanpred) and NetCTL-1.2 [61]. Results are presented as the individual outputs of NetCTLpan-1 and NetMHCpan-2.2 (which have been reported as having a higher accuracy [57, 59]) or as grouped outputs for the other programs used. Positive results generated by NetCTLpan were assigned ‘*’ if the putative epitopes were correctly processed (proteasome and transporters associated with antigen processing [TAP]) and bound to the MHC. For all other programs, positive results were classified based on binding strength to the MHC, either as strong or weak binders (‘SB’ or ‘WB’ , respectively) based on the author-recommended cut-offs.

## Availability of supporting data

The data sets supporting the results of this article are included in Tables 1, 2, 3, 4 and 5 and Fig. 1 within the article, and in its Additional files 1, 2 and 3. The 15-mer class II-binding peptides listed in Table 5 were originally derived from Supplementary Table S1 in ref. [18], and allocated to Mtb32A or Mtb39A by the authors of the latter publication.

### Ethics statement

The authors declare that this study does not involve any ethical issues, since no animal or human experiments were performed as part of the research for this manuscript.

## Additional files

Additional file 1:
**Predicted HLA class II binding peptides in Mtb72F.** (XLSX 15 kb) Additional file 2:
**Frequency of alleles without predicted binding peptides in the populations assessed.** (PDF 66 kb) Additional file 3:
**Predicted** **9-mer** **HLA class I peptide binding regions in Mtb72F.** (XLSX 13 kb)
