# Supplementary material for: Sequence conservation analysis and in silico human leukocyte antigen-peptide binding predictions for the Mtb72F and M72 tuberculosis candidate vaccine antigens
Source: BMC Immunol. 2015 Oct 22;16:63. doi: 10.1186/s12865-015-0119-7 (PMC4619029; doi:10.1186/s12865-015-0119-7)
Supplement: Additional file 2: — Frequency of alleles without predicted binding peptides in the populations assessed. (PDF 66 kb) [file 12865_2015_119_MOESM2_ESM.pdf]

Additional File 2. Frequency of alleles without predicted binding peptides in the populations assessed.

Allele	Population, Country/region	Allele Freq# (%)	Sample Size (N)	Ref. no. $
DRB1*03:02	Aka Pygmy, Centr. African Rep.	0.00	93	[1]
DRB1*03:02	Urumqi Uyghur, China	0.00	57	[2]
DRB1*03:02	Wuhan, China	0.40	121	[3]
DRB1*03:02	Bantu, Kinshasa, Congo	3.30	90	[1]
DRB1*03:02	Asutuare, Ghana	17.20	429	[4]
DRB1*03:02	Kayastha, NE India	0.30	190	[5]
DRB1*03:02	Lachung, NE India	0.00	58	[5]
DRB1*03:02	Mathur, NE India	0.00	155	[5]
DRB1*03:02	Mech, NE India	0.00	63	[5]
DRB1*03:02	Rajbanshi, NE India	0.00	98	[5]
DRB1*03:02	Rastogi, NE India	1.50	196	[5]
DRB1*03:02	Shia, NE India	0.00	190	[5]
DRB1*03:02	Sunni, NE India	0.00	188	[5]
DRB1*03:02	Vaish, NE India	0.00	198	[5]
DRB1*03:02	General pop., Rwanda	4.10	280	[6]
DRB1*03:02	Hutu and Tutsi, Kigali, Rwanda	4.10	281	[7]
DRB1*03:02	Dakar, Senegal	3.30	112	[8]
DRB1*03:02	Native population, S. Africa	9.70	200	[9]
DRB1*03:02	Venda, Limpopo, S. Africa	9.00	117	[10]
DRB1*03:02	Shona, Harare, Zimbabwe	6.60	230	[11]
DRB1*03:02:01	General pop., Cameroon	6.70	126	[12]
DRB1*03:02:01	Stem cell donors, Nigeria	5.06	258	[13]
DRB1*03:02:02	Maratha, Mumbai, India	0.40	91	[14]
DRB1*16:04	Aka Pygmy, Centr. African Rep.	0.00	93	[1]
DRB1*16:04	Urumqi, Uyghur, China	0.00	57	[2]
DRB1*16:04	Han, Yunnan, China	0.40	129	[15]
DRB1*16:04	Naxi, Yunnan, China	0.00	118	[15]
DRB1*16:04	Bantu, Kinshasa, Congo	0.00	90	[1]
DRB1*16:04	Dravidian, Tamilnadu, S India	0.60	156	[16]
DQA1*02:01-DQB1*02:01	-	-	-	-
DPA1*02:02-DPB1*05:01	-	-	-	-
DRB1*07:01-DQA1*02:01-DQB1*02:01	Urumqi Han, China	10.20	59	[17]
DRB1*07:01-DQA1*02:01-DQB1*02:01	Urumqi Kazak, China	8.30	42	[17]
DRB1*07:01-DQA1*02:01-DQB1*02:01	Haut-Ogooue Dienga, Gabon	4.90	167	[18]
DRB1*07:01-DQA1*02:01-DQB1*02:01	Kayastha, NE India	12.00	190	[5]
DRB1*07:01-DQA1*02:01-DQB1*02:01	Mathur, NE India	14.80	155	[5]
DRB1*07:01-DQA1*02:01-DQB1*02:01	Rastogi, NE India	9.40	196	[5]
DRB1*07:01-DQA1*02:01-DQB1*02:01	Vaish, NE India	12.60	198	[5]
DRB1*07:01-DQA1*02:01-DQB1*02:01	Shia, NE India	6.20	190	[5]
DRB1*07:01-DQA1*02:01-DQB1*02:01	Sunni, NE India	6.20	188	[5]
DRB1*07:01-DQA1*02:01-DQB1*02:01	Lachung, NE India	7.00	58	[5]
DRB1*07:01-DQA1*02:01-DQB1*02:01	Mech, NE India	4.20	63	[5]
DRB1*07:01-DQA1*02:01-DQB1*02:01	Rajbanshi, NE India	8.60	98	[5]
DRB1*07:01-DQA1*02:01-DQB1*02:01	Uttar Pradesh, N India	12.80	202	[19]
DRB1*07:01-DQA1*02:01-DQB1*02:01-DPB1*02:01	Han, Canton, China	1.40	264	[20]
DRB1*07:01-DQA1*02:01-DQB1*02:01-DPB1*03:01	Han, Canton, China	1.10	264	[20]
DRB1*07:01-DQA1*02:01-DQB1*02:01-DPB1*17:01	Han, Canton, China	1.90	264	[20]

Information sourced from the 'Allele*frequencies in Worldwide Populations' database [21] http://www.allelefrequencies.net, accessed 26 January 2015. # Allele frequency: Total number of copies of the allele in the population sample (alleles / 2n), in percentages. '-', no results given in database. $, Full references provided below.

References Additional File 2:

1. Renquin J, Sanchez-Mazas A, Halle L, Rivalland S, Jaeger G, Mbayo K, et al. HLA class II polymorphism in Aka Pygmies and Bantu Congolese and a reassessment of HLA-DRB1 African diversity. Tissue Antigens. 2001;58:211-22.
2. Mizuki N, Ohno S, Ando H, Sato T, Imanishi T, Gojobori T, et al. Major histocompatibility complex class II alleles in an Uygur population in the Silk Route of Northwest China. Tissue Antigens. 1998;51:287-92.
3. Ferencik S, Gong F, Grosse-Wilde H. HLA gene frequencies: Chinese (Wuhan) Normal. In: Gjertson DW, Terasaki PI, editors. HLA-1998. Los Angeles, CA: UCLA Tissue Typing Laboratory; 1998. p. 202-3.
4. Yamazaki A, Yasunami M, Ofori M, Horie H, Kikuchi M, Helegbe G, et al. Human leukocyte antigen class I polymorphisms influence the mild clinical manifestation of *Plasmodium falciparum* infection in Ghanaian children. Hum Immunol. 2011;72:881-8.
5. Agrawal S, Srivastava SK, Borkar M, Chaudhuri TK. Genetic affinities of north and northeastern populations of India: inference from HLA-based study. Tissue Antigens. 2008;72:120-30.
6. Tang J, Naik E, Costello C, Karita E, Rivers C, Allen S, et al. Characteristics of HLA class I and class II polymorphisms in Rwandan women. Exp Clin Immunogenet. 2000;17:185-98.
7. Tang J, Allen S, Karita E, Kaslow RA. Hutu and Tutsi from Kigali. In: Hansen JA, editors. Immunobiology of the Human MHC: 13th International Histocompatibility Workshop and Conference. Seattle, WA, USA: IHWG Press; 2006. p. 586-7.
8. Andrien M, Defleur V, Vandercruys M, Dupont E. Senegalese normal. In: Terasaki PI, Gjertson DW, editors. HLA 1997. Los Angeles, CA, USA: UCLA Tissue Typing Laboratory; 1997. p. 178.
9. Paximadis M, Mathebula TY, Gentle NL, Vardas E, Colvin M, Gray CM, et al. Human leukocyte antigen class I (A, B, C) and II (DRB1) diversity in the black and Caucasian South African population. Hum Immunol. 2012;73:80-92.
10. Lombard Z, Dalton DL, Venter PA, Williams RC, Bornman L. Association of HLA-DR, -DQ, and vitamin D receptor alleles and haplotypes with tuberculosis in the Venda of South Africa. Hum Immunol. 2006;67:643-54.
11. Louie L. Immunobiology of the Human MHC. In: Hansen JA, editors. Proceedings of the 13th International Histocompatibility Workshop and Conference. Seattle: IHWG Press; 2006.
12. Pimtanothai N, Hurley CK, Leke R, Klitz W, Johnson AH. HLA-DR and -DQ polymorphism in Cameroon. Tissue Antigens. 2001;58:1-8.
13. Adebiyi S. Unpublished data.
14. Shankarkumar U, Pawar A, Ghosh K, Bajpai S, Pazare A. Human leucocyte antigen class II DRB1 and DQB1 associations in human immunodeficiency virus-infected patients of Mumbai, India. Int J Immunogenet. 2010;37:199-204.
15. Shi L, Xu SB, Ohashi J, Sun H, Yu JK, Huang XQ, et al. HLA-A, HLA-B, and HLA-DRB1 alleles and haplotypes in Naxi and Han populations in southwestern China (Yunnan province). Tissue Antigens. 2006;67:38-44.
16. Rajeswari DN, Selvaraj P, Raghavan S, Jawahar MS, Narayanan PR. Influence of HLA-DR2 on perforin-positive cells in pulmonary tuberculosis. Int J Immunogenet. 2007;34:379-84.
17. Mizuki M, Ohno S, Ando H, Sato T, Imanishi T, Gojobori T, et al. Major histocompatibility complex class II alleles in Kazak and Han populations in the Silk Route of northwestern China. Tissue Antigens. 1997;50:527-34.
18. Migot-Nabias F, Fajardy I, Danze PM, Everaere S, Mayombo J, Minh TN, et al. HLA class II polymorphism in a Gabonese Banzabi population. Tissue Antigens. 1999;53:580-5.
19. Agrawal S, Khan F, Bharadwaj U. Human genetic variation studies and HLA class II loci. Int J Immunogenet. 2007;34:247-52.
20. Trachtenberg E, Vinson M, Hayes E, Hsu YM, Houtchens K, Erlich H, et al. HLA class I (A, B, C) and class II (DRB1, DQA1, DQB1, DPB1) alleles and haplotypes in the Han from southern China. Tissue Antigens. 2007;70:455-63.
21. Gonzalez-Galarza FF, Christmas S, Middleton D, Jones AR. Allele frequency net: a database and online repository for immune gene frequencies in worldwide populations. Nucleic Acids Res. 2011;39:D913-D919.
